# lncRNA-based prognostic model for pancreatic cancer centered on the TME with exploratory LLPS connections

**DOI:** 10.3389/fonc.2026.1753321

**Published:** 2026-01-23

**Authors:** Yaqing Wei, Xiguang Sun, Changjun Ding, Yifei Wang, Zheran Lu, Chenhui Zhang, Hao Yao, Hao Huang

**Affiliations:** 1 Department of Hepatopancreatobiliary Surgery, The Second Hospital of Tianjin Medical University, Tianjin, China; 2 Tianjin Medical University, Tianjin, China; 3 RWTH Aachen University, Aachen, North Rhine-Westphalia, Germany

**Keywords:** LLPS, LncRNA - long noncoding RNA, pancreatic cancer, prognosis, TME (tumor microenvironment)

## Abstract

**Introduction:**

Liquid-Liquid Phase Separation (LLPS), tumor microenvironment (TME), and long non-coding RNA (lncRNA) all have varying degrees of influence on the expression regulation of tumors. However, research on the association of these three in pancreatic cancer (PC) still requires further exploration. This study seeks to establish the relationships among these three themes through bioinformatics and to identify biomarkers that can predict the prognosis of PC patients.

**Methods:**

Data sets from The Cancer Genome Atlas (TCGA) and International Cancer Genome Consortium (ICGC) are obtained from the UCSC platform. lncRNAs associated with the LLPS and TME gene sets are screened, and model lncRNAs are identified through comprehensive analysis conducted with least absolute shrinkage and selection operator (LASSO) regression and cox proportional hazards (COX) regression. Additionally, the predictive efficacy of the model lncRNAs is validated through multiple databases and cohorts. Furthermore, the expression of the model lncRNAs is validated at a biological level.

**Results:**

A comprehensive analysis establishes an optimal combination consisting of 5 lncRNAs. The Kaplan–Meier curves and receiver operating characteristic (ROC) curves for each cohort demonstrates the effectiveness of the model lncRNAs characteristics. Additionally, the COX regression analysis of clinical characteristics and the analysis of mutation data further indicates the stability of the model lncRNAs. Furthermore, the expression levels of model lncRNAs in cell lines are consistent with the analysis results.

**Conclusion:**

The model lncRNAs identified in this study, which are correlated with LLPS and TME, demonstrate significant potential as independent biomarkers for predicting the prognosis of PC patients.

## Introduction

PC has been increasing in both incidence and mortality rates each year. It has been reported that PC ranks 12th in incidence among all cancers and 6th in cumulative mortality rates (1). It is project that by 2030, PC will become the second leading cause of cancer-related deaths in the United States (2). Studies indicate that the lifetime risk of developing PC worldwide is up to 0.89% (95%CI: 0.88–0.89), and the median overall survival (OS) among all stages of the disease is only 4 months (3–5). This severe treatment burden imposes a heavy burden on socioeconomic development, making it urgent to explore potential prognostic biomarkers to guide treatment for PC.

Previous studies have shown that the assessment of clinical risk factors for PC prognosis is unsatisfactory. Known risk factors do not sufficiently explain the development of PC (6, 7). However, in recent years, lncRNA has garnered widespread attention in oncology research (8). lncRNA influences tumor occurrence and progression through various mechanisms, including gene expression, post-transcriptional regulation, and cell proliferation (9, 10). Particularly in PC, abnormal expression of lncRNA is closely associated with tumor aggressiveness, metastasis, and prognosis (11, 12). Additionally, the characteristics of the PC microenvironment are distinct, including hypoxia and high fibrosis (13, 14). lncRNA participates in the construction of this network by regulating immune cells and cancer-associated fibroblasts within the TME (15, 16). Notably, lncRNA can also influence the expression of the TME by regulating the extracellular matrix, metabolic status, and intercellular signaling (17).

It is worth noting that the close relationship between lncRNA and LLPS during tumor progression is one of the important factors affecting the TME (18, 19). lncRNA can form condensates in the nucleus through LLPS, and these condensates not only participate in the regulation of gene transcription but may also act as signal integrators to respond to changes in the microenvironment (20, 21). Normal biomolecular condensates ensure fundamental cellular functions, while their anomalous forms can lead to cellular dysfunction and potential tumor (22). Studies have demonstrated that LLPS plays a crucial role in regulating tumor proliferation and metastasis (23, 24). A significant number of lncRNAs are localized on chromatin and often form RNA-clouds within specific nuclear regions to regulate gene expression. As major components of various membraneless structures, such as nucleolus and paraspeckles, lncRNAs are frequently imbalanced in cancer (25). Recent research has shown that certain lncRNA subdomains can selectively combine to proteins like NONO/SFPQ, which dynamically oligomerize and recruit additional proteins through LLPS (26). This process not only participates in gene expression regulation, RNA processing, and cellular stress responses but is also highly sensitive to cellular states and environmental signals (27, 28). In summary, this study will anchor biomarkers at the mechanistic level of tumors, screening lncRNA based on the LLPS and TME gene sets to give a reference for the prognosis of PC.

## Methods

### Data acquisition and integration

The data included in this study originates from the TCGA and ICGC datasets on the UCSC platform. 165 tumor samples and 171 normal tissue samples originate from the GTEx-TCGA dataset. This dataset is employed for model construction and serves as an internal validation dataset. To enhance the stability of the model, Researchers also include 90 pancreatic tumor samples sourced from the ICGC dataset, which serve as an external validation dataset. Additionally, differential expression analysis of the GTEx-TCGA gene matrix is conducted with the R language software version 4.2.3 (log_2_FC > 1.0, FDR< 0.05, *P-*value< 0.05). Overall, a total of 5, 901 genes and 200 lncRNAs are identified as significantly differentially expressed.

### Confirm the theme-related target gene and potential lncRNA

Based on the GeneCards search engine, gene sets are retrieved with the keywords ‘TME’ and ‘LLPS’. Following this, the intersection of these two gene sets is constructed. This intersected gene set is then combined with the 5, 901 differentially expressed genes to identify those that are associated with both themes. Subsequently, the limma R package is applied to perform a correlation analysis between the identified lncRNAs and the intersected genes. As a result, 138 lncRNAs that are linked to both ‘TME’ and ‘LLPS’ are confirmed as potential lncRNAs for further analysis.

### Model construction and cohort grouping

To test the effectiveness of the model lncRNA, 165 tumor samples from the GTEx-TCGA dataset are randomly divided into two independent cohorts: a training cohort and a validation cohort. The optimal model is constructed with COX regression, and LASSO regression. To assess the performance of the model lncRNA and reduce the risk of overfitting, 10-fold cross-validation is conducted along with 1, 000 random perturbations applied to the training cohort. The formula is then applied to calculate the risk score, where lncRNA^k^= coef(lncRNA^k^)× expr(lncRNA^k^), with “coef” representing the survival coefficient and “expr” indicating the gene expression level. Based on the median risk score, both the training and validation cohort, as well as the external validation dataset, are all classified into low-risk and high-risk subgroups for further analysis.


$$\text{risk}\;\text{score}=\sum_{\text{k}=1}^{\text{n}}\left(\text{lncRN}{\text{A}}^{\text{k}}\right)\;$$

### Assess the survival prediction efficacy of model lncRNA

The researchers conduct internal validation by comparing survival status and survival curves of risk subgroups within the training and validation cohorts. To further evaluate the predictive efficacy of model lncRNA in the internal dataset, time-dependent ROC curves and clinical characteristics ROC curves are employed. Building on this evaluation, survival analyses and time-dependent ROC curves for the risk subgroups are performed on the entire TCGA dataset. These analyses are also conducted in an external dataset to illustrate the efficacy of the model lncRNA. Furthermore, researchers compare survival differences in clinical characteristics, such as age, gender, tumor grade, and tumor stage, between low-risk and high-risk subgroups. Finally, the reliability of risk scores is confirmed through the construction of univariate and multivariate COX regression analysis, which develops the predictive capability of the model lncRNA.

### Construction of mutation data analysis

To further illustrate the efficacy of the model lncRNA, this study extracts mutation data from samples in the TCGA database. Researchers perform correlation analyses to reveal differences in mutation data between the low-risk and high-risk subgroups. Following this, the study categorizes the samples into two groups based on the median tumor mutation burden (TMB) coefficient: low-TMB (L-TMB) and high-TMB (H-TMB). Subsequently, researchers construct survival analysis curves for the mutation subgroups. Finally, researchers correlate the mutation subgroups with the risk subgroups to conduct survival analysis. This analysis strengthens the evidence for the model lncRNAs effectiveness and stability.

### Model lncRNAs expression validation in cell lines

In this study, four cell lines—hTERT-HPNE (HPDE), PANC-1, ASPC-1, and MIA-PACA-2—are selected for qPCR experiments. The primer and housekeeping gene sequences applied in the research can be found in **Supplementary Data Sheet 9**. Each gene is conducted in triplicate and repeated three times in different cell lines. The results are averaged, and the standard deviation is calculated to assess variability. Relative expression is quantified using the 2^-ΔΔCT^ method, and statistical analysis is performed with a t-test. Finally, the results are visualized with Prism software version 9.5.1.

## Results

### Selection of target gene and model lncRNA

**Figure 1:**
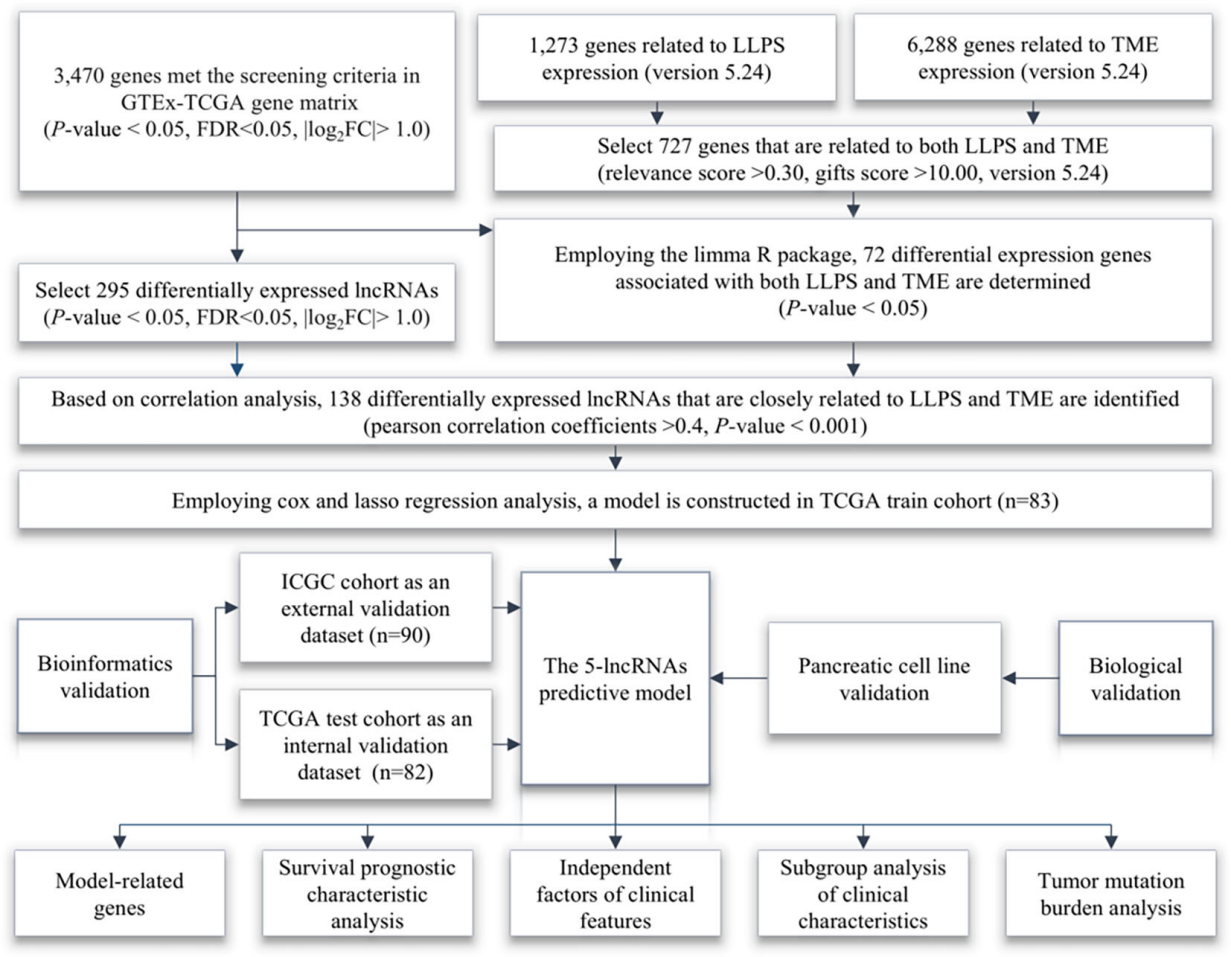
Research flowchart. The arrows indicate the steps of research progress.

**Figure 2:**
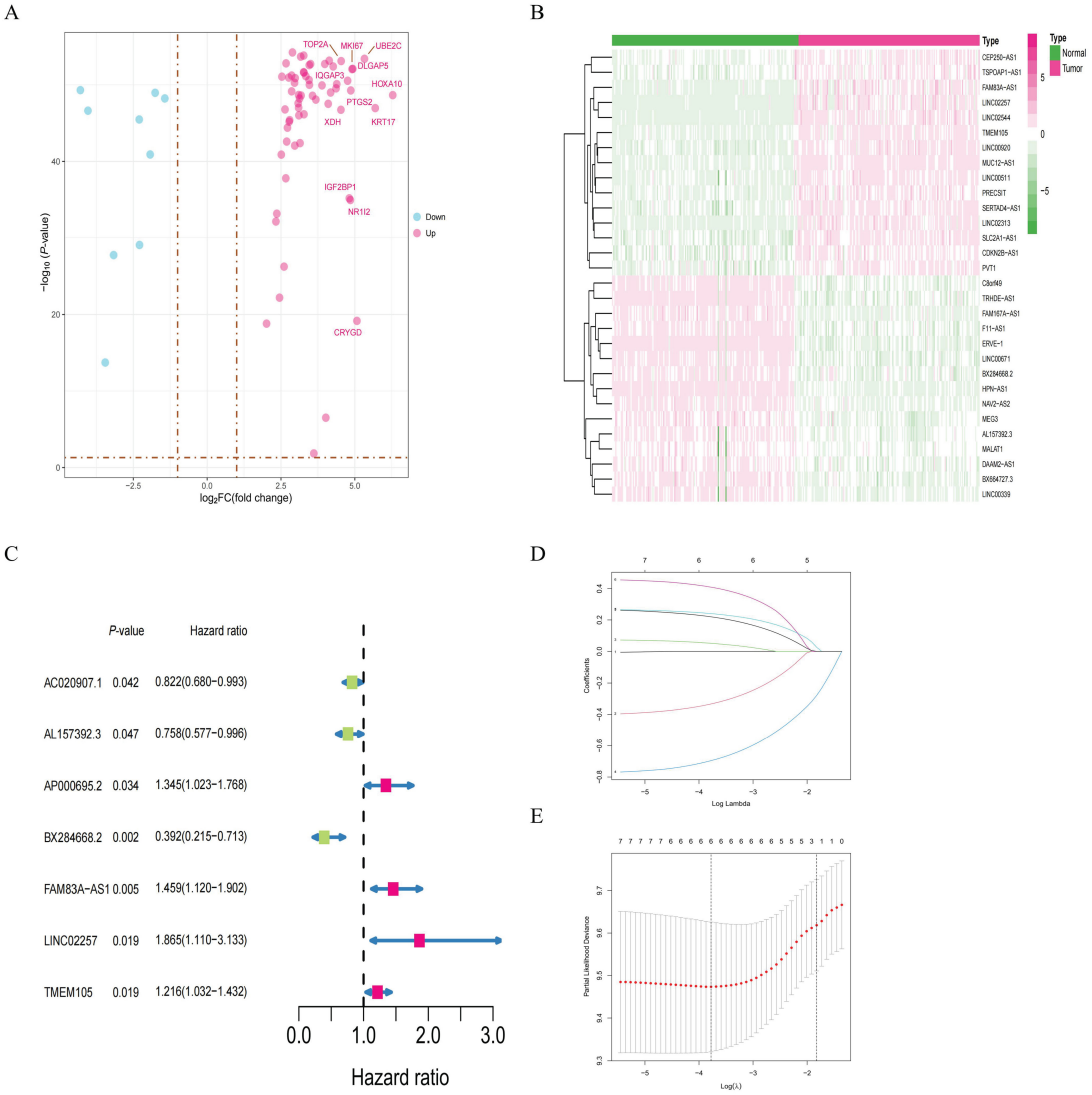
Identification of target genes and lncRNAs. **(A)** Volcano plot of 72 target genes. **(B)** Heatmap of the top 30 target genes with the highest correlation from the correlation analysis results. **(C)** Seven potential signature lncRNAs selected through univariate COX regression. **(D)** Coefficient path diagram from LASSO regression. **(E)** Likelihood ratio test plot for the model from LASSO regression.

**Figure 3:**
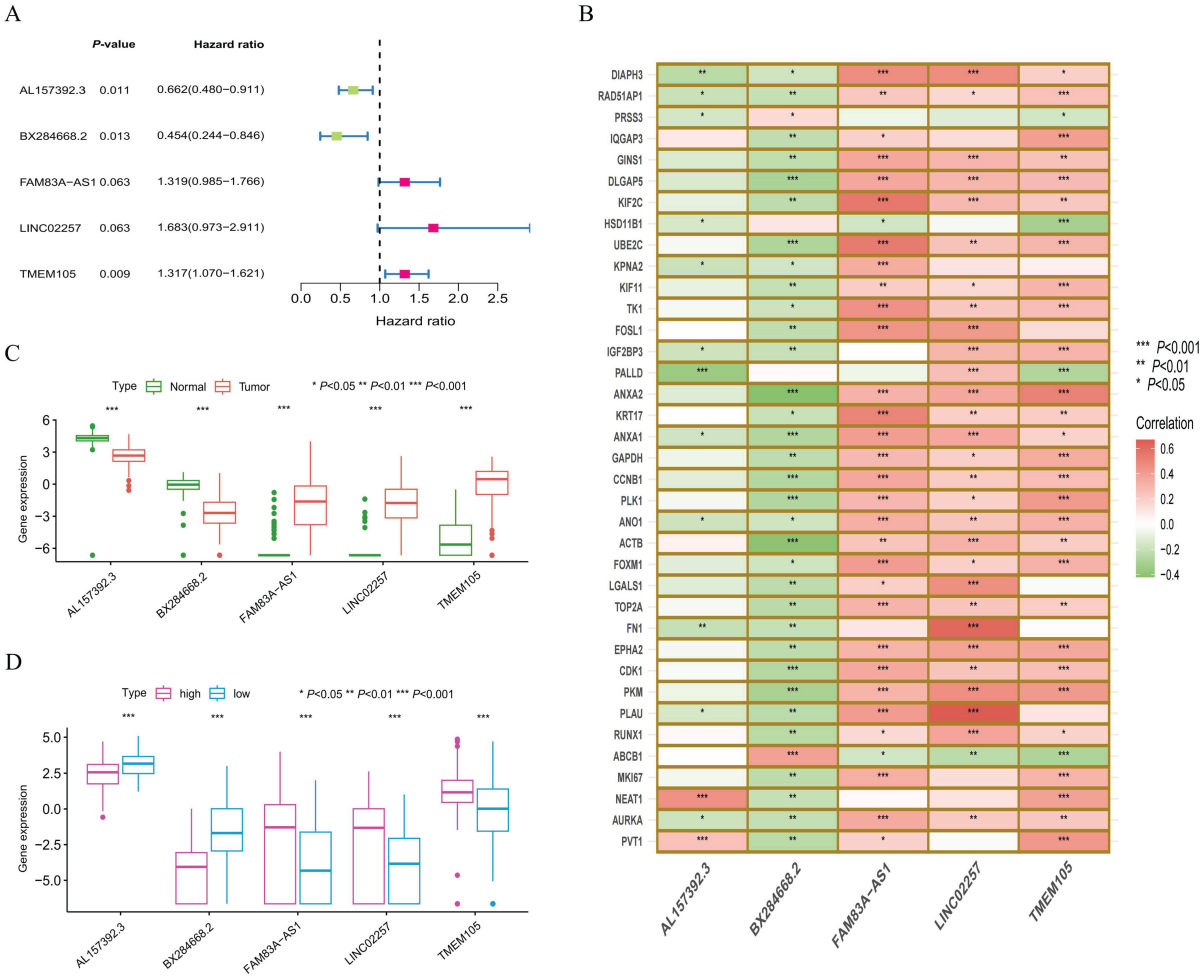
Correlation analysis of model lncRNAs. **(A)** The optimal model combinations established by 5-lncRNAs through multivariate COX analysis. **(B)** Heatmap of target genes significantly associated with model lncRNAs. Indicating that (*, **, and *** represent p-values less than 0.05, 0.01, and 0.001, respectively). **(C)** Expression levels of model lncRNAs in normal and tumor samples. **(D)** Expression levels of model lncRNAs in risk subgroups.

The research flowchart for this study is shown in **Figure 1**. Through the analysis of sample data from the GTEx-TCGA dataset, researchers discover 3469 genes that are differentially expressed (**Supplementary Data Sheet 1**). We subsequently obtained 727 theme genes from the GeneCards website (version 5.24) with gifts score greater than 10 and a relevance score greater than 0.30 (**Supplementary Data Sheet 2**). After intersecting the differentially expressed genes with the theme genes, researchers discover 72 target genes (**Figure 2A**). From the correlation analysis results, researchers discover 138 lncRNAs linked to target genes, prompting the development of a heatmap showing the top 30 lncRNAs (**Figure 2B**; **Supplementary Data Sheet 3**). The training and validation cohorts are validated as independent datasets based on the chi-square method (**Table 1**). Analyzing the sample data from the training cohort, researchers initially identify 7 lncRNAs through univariate COX regression analysis (**Figure 2C**). To validate the result, researchers subsequently perform LASSO analysis, conducting 1, 000 random cycles (**Figures 2D, E**). Finally, through multivariate COX regression analysis, 5 lncRNAs are selected as model lncRNAs (**Figure 3A**).

**Table 1:** Basic information of the random cohort.

Clinical indicators	Classification	Total	Validation	Training	*P*-value
Age (Year)	<=65	84	41(51.25%)	43(53.09%)	0.9399
	>65	77	39(48.75%)	38(46.91%)	
Gender	Female	74	36(45.00%)	38(46.91%)	0.9319
	Male	87	44(55.00%)	43(53.09%)	
Grade	1	25	11(13.75%)	14(17.28%)	0.4424
	2	89	48(60.00%)	41(50.62%)	
	3	46	20(25.00%)	26(32.10%)	
	4	1	1(1.25%)	0(0%)	
Stage	I	17	10(12.50%)	7(8.64%)	0.6011
	II	137	68(85.00%)	69(85.19%)	
	III	3	1(1.25%)	2(2.47%)	
	IV	4	1(1.25%)	3(3.70%)	
T staging	T_1_	6	5(6.25%)	1(1.23%)	0.3240
	T_2_	19	8(10.00%)	11(13.58%)	
	T_3_	133	66(82.50%)	67(82.72%)	
	T_4_	3	1(1.25%)	2(2.47%)	
N staging	N_0_	46	24(30.00%)	22(27.16%)	0.8225
	N_1_	115	56(70.00%)	59(72.84%)	

### Establishment and expression differential analysis of model lncRNA

The correlation analysis based on the model lncRNAs and differently expressed genes show that the model lncRNAs are related to many co-related genes in LLPS and TMU (**Figure 3B**). Additionally, researchers assess the expression differences of model lncRNAs between tumor and normal samples, as well as within the subgroups of high-risk and low-risk (**Figures 3C, D**). The results indicate significant differences in the expression of model lncRNAs among samples. Furthermore, the results of model RNA are consistent with the multivariate COX result, with *AL157392.3* and *BX284668.2* as low-risk genes, while the other model lncRNAs are high-risk genes.

### Internal dataset survival analysis

**Figure 4:**
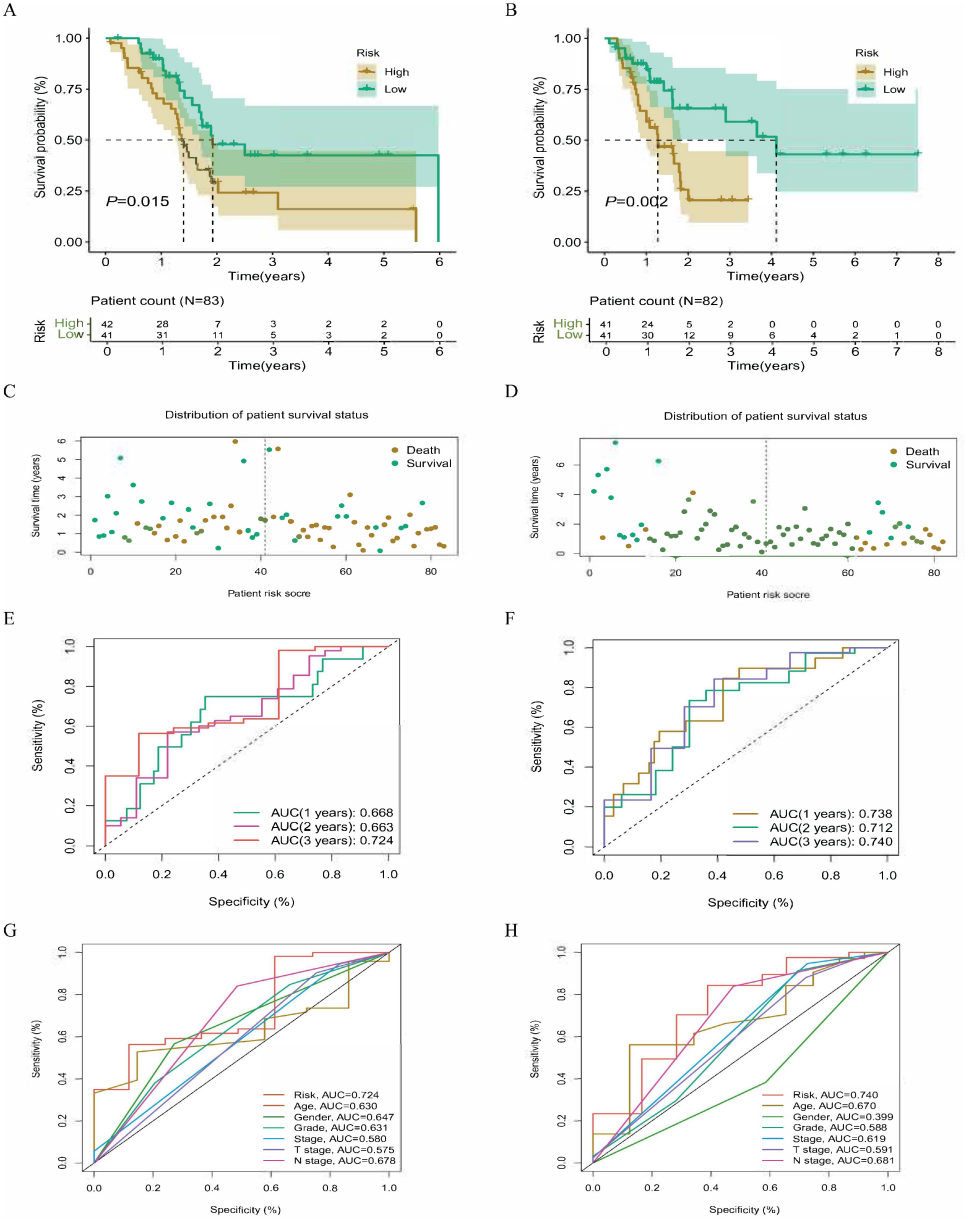
Survival analysis of internal cohort samples. Survival curves for training cohort and validation cohort samples **(A, B)**; Distribution of survival status for training cohort and validation cohort **(C, D)**. The ROC curve distribution for training cohort and validation cohort samples **(E, F)**. The ROC curve distribution for clinical characteristics of training cohort and validation cohort **(G, H)**.

**Figure 5:**
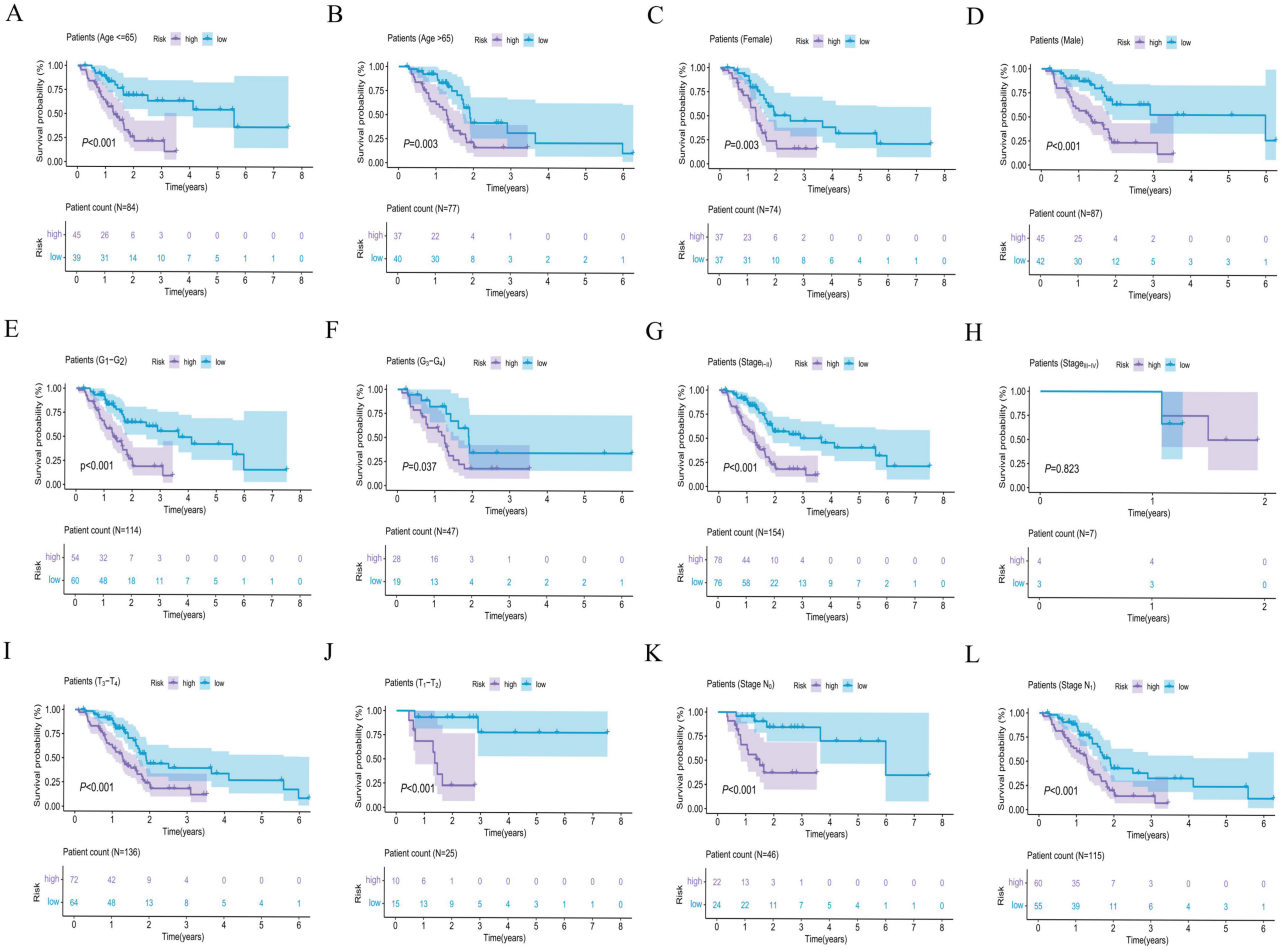
Survival analysis curves for different clinical groups. Risk subgroup survival curves constructed based on clinical characteristics such as patient age **(A, B)**, gender **(C, D)**, tumor stage **(E, F)**, tumor grade **(G, H)**, T staging **(I, J)**, and N staging **(K, L)**.

The analysis of risk subgroups reveals significant differences in survival outcomes in both the training and validation cohorts (**Supplementary Data Sheet 4**, **5**). Specifically, the prognosis for the low-risk subgroup is found to be significantly longer than that for the high-risk subgroup (**Figures 4A, B**). In both cohorts, the assessment of risk status indicates that the number of patients deaths clearly increases with the risk scores (**Figures 4C, D**). The area under the time-dependent ROC curve (AUC) for both datasets surpass 0.66, effectively demonstrating the prediction capability of model lncRNAs (**Figures 4E, F**). Furthermore, ROC curves for clinical characteristics indicate that the risk scores is superior to other clinical characteristics as an indicator of prognosis (AUC > 0.7) (**Figures 4G, H**). To further evaluate the predictive performance of the model, this study assesses the survival time in risk subgroups across various clinical categories (age, sex, grade, and stage, etc). The results highlight significant differences between the low-risk and high-risk subgroups in most clinical categories, thereby further confirming the predictive accuracy of the model lncRNAs (**Figures 5A–L**).

### External database validation

**Figure 6:**
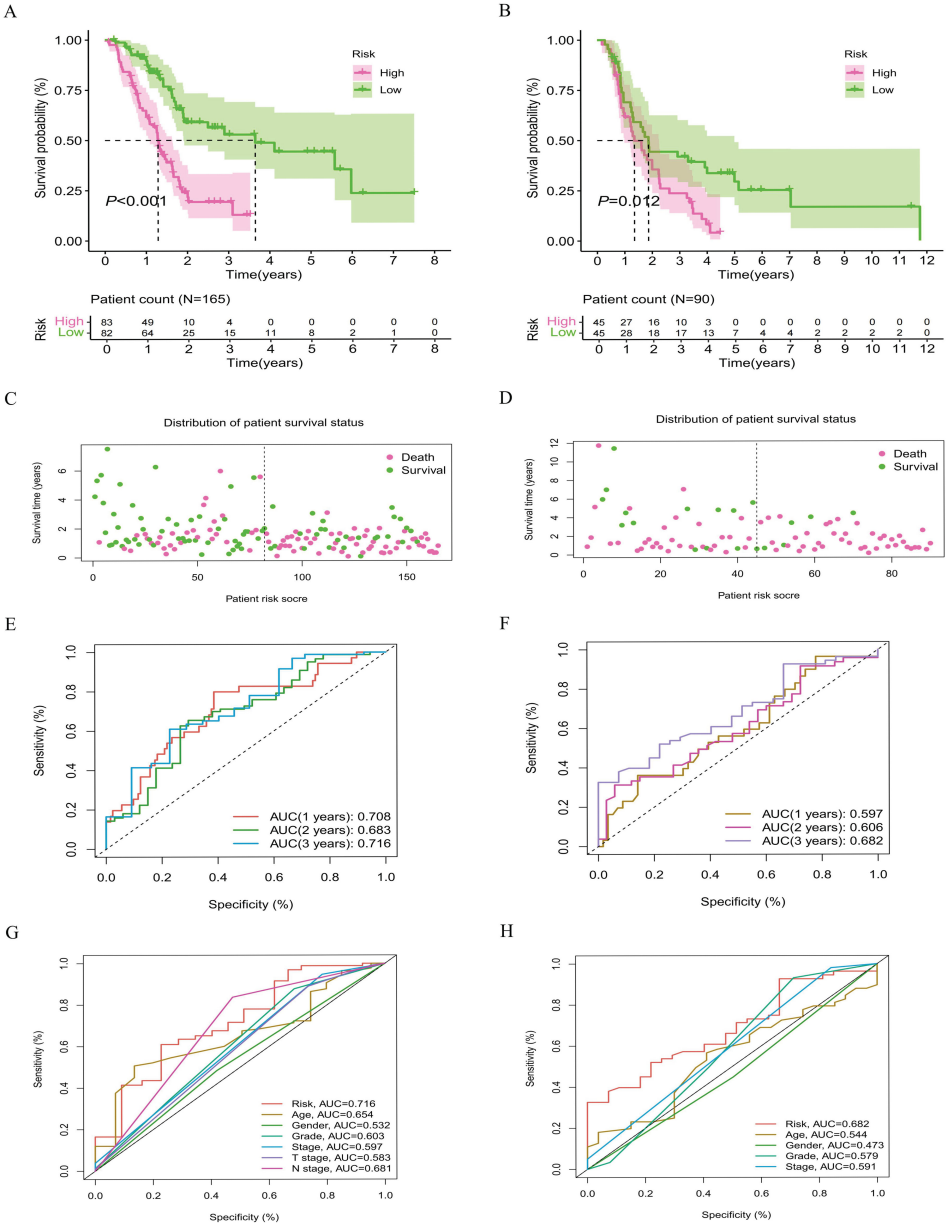
Survival analysis of samples from different databases. Survival curves of samples from TCGA and ICGC database **(A, B)**; Distribution of survival status for samples from TCGA and ICGC database **(C, D)**. ROC curve distribution for samples from TCGA and ICGC database **(E, F)**. ROC curve distribution of clinical characteristics for samples from TCGA and ICGC database **(G, H)**.

This study further analyzes the differences in risk subgroups among samples from the TCGA and ICGC databases (**Supplementary Data Sheet 6**, **7**). Survival curves indicate that the high-risk subgroup has a shorter survival time compared to the low-risk subgroup (**Figures 6A, B**). Additionally, the assessment of survival status clearly demonstrates that there are fewer patients surviving in the high-risk subgroup (**Figures 6C, D**). The AUCs based on samples from both databases are basically greater than 0.6 (**Figures 6E, F**). While the time-dependent ROC curve within the ICGC dataset shows the lowest value of 0.597 during the first year, it is noteworthy that the predictive accuracy of the model exhibits an overall upward trend over time. Moreover, the AUC for clinical characteristics based on samples from both datasets exceeds 0.68, further confirming the predictive efficacy of the model lncRNAs (**Figures 6G, H**). Through verification analysis across different databases, this study evaluates the survival prediction capability of the model lncRNAs from multiple perspectives.

### Assessment of clinical features of model lncRNAs

**Figure 7:**
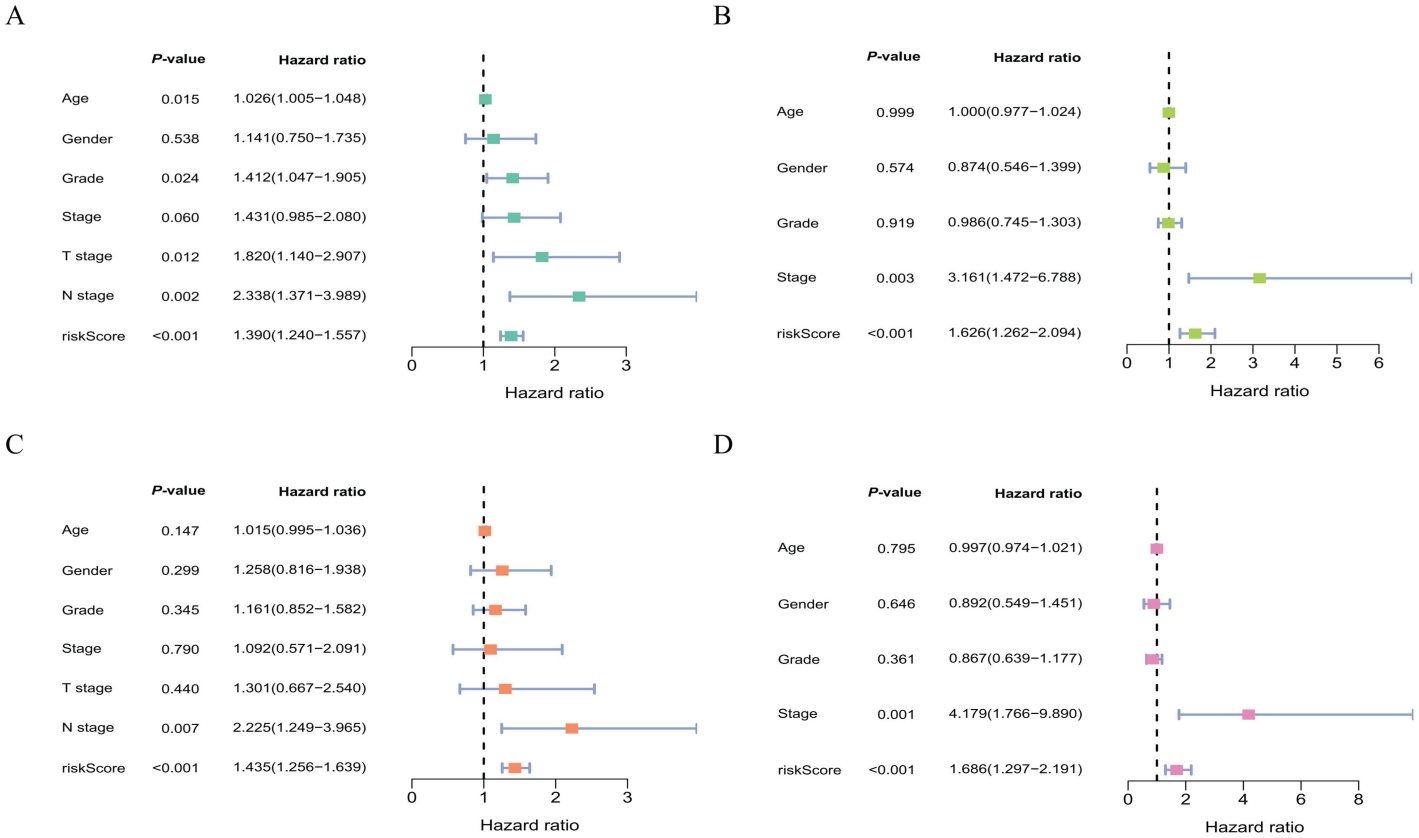
Assessment of clinical characteristics from samples in different databases. Univariate COX regression analysis of clinical characteristics from samples in the TCGA and ICGC database **(A, B)**; multivariate COX regression analysis of clinical characteristics from samples in the TCGA and ICGC database **(C, D)**.

To further validate the predictive efficacy of the model lncRNAs for prognosis, this study conducts univariate and multivariate COX regression analyses employing clinical characteristics from samples in the TCGA and ICGC datasets. Both univariate and multivariate COX analyses provide consistent results, indicating that the risk score is significantly associated with patient prognosis (**Figures 7A–D**). This demonstrates that the risk score can serve as an independent risk factor for clinical prognosis assessment, thereby further highlighting the stability of the model lncRNAs in evaluating prognostic efficacy. Additionally, the analysis indicates that tumor stage in ICGC dataset and tumor N staging in TCGA dataset also serve as independent factors.

### Mutation differences in risk subgroups

**Figure 8:**
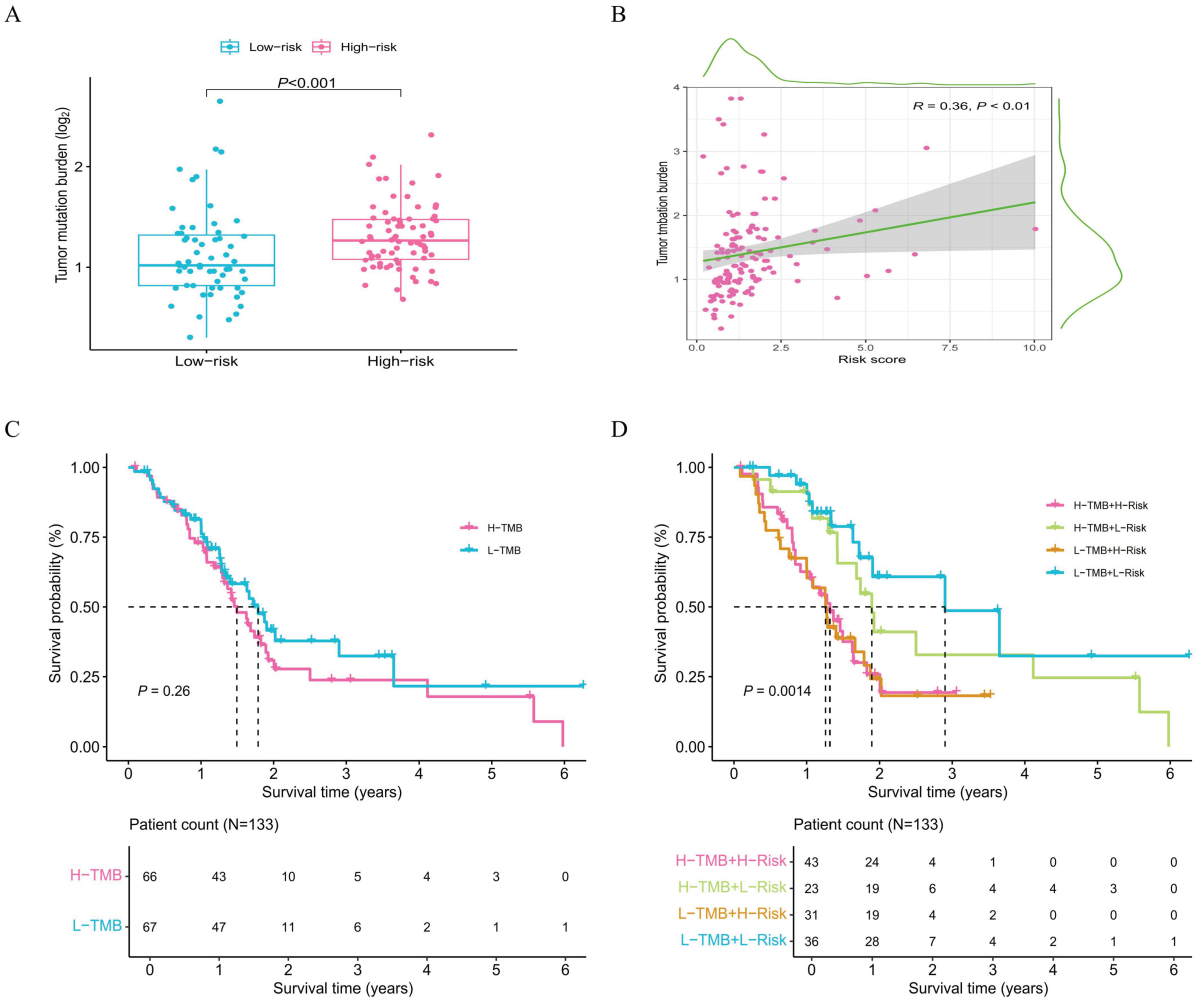
Survival analysis of risk subgroups associated with mutation data. **(A)** Comparison of TMB differences in risk subgroups; **(B)** Correlation analysis between risk scores and TMB value. **(C)** Survival analysis curve for mutation subgroups. **(D)** Survival analysis curves for samples in the mutation and risk subgroup.

To further compare the differences between the risk subgroups, this study analyzes mutation data of the samples (**Supplementary Data Sheet 8**). Researchers obtain TMB values for the samples from the TCGA database. The analysis indicates a statistically significant difference in TMB values between the two risk subgroups, demonstrating that mutations vary between them (**Figure 8A**). Additionally, the correlation analysis result demonstrates a significant positive correlation between risk scores and TMB values (**Figure 8B**). Specifically, this analysis shows that as the risk score increases, the TMB value also rises significantly. Furthermore, survival analysis results reveal clear differences in survival time in the subgroups (**Figures 8C, D**). This finding further highlights the sensitivity and stability of risk genes in predicting the prognosis status of the samples.

### qPCR validation of model lncRNAs expression

**Figure 9:**
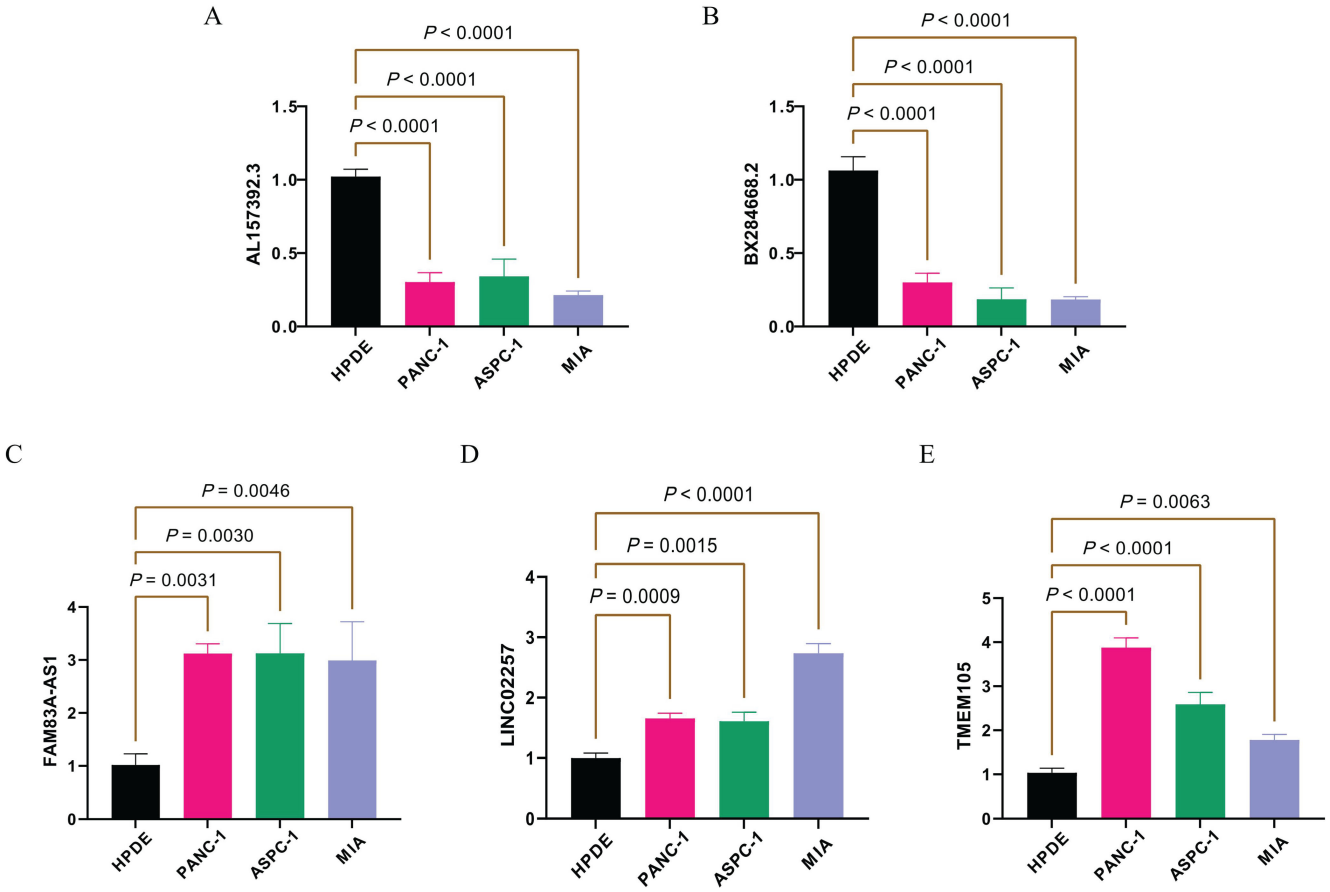
The model lncRNA expression levels in different pancreatic cell lines. The expression of model lncRNA AL157392.3 **(A)**, BX284668.2 **(B)**, FAM83A-AS1 **(C)**, LINC02257 **(D)**, and TMEM105 **(E)** in different pancreatic cell lines.

The study analyzes the expression levels of model lncRNAs among different cell lines (**Supplementary Data Sheet 9**). Specifically, the gene expression levels of the HPDE normal pancreatic ductal tissue cell line are selected as a reference. The protective genes *AL157392.3* and *BX284668.2* demonstrate significantly higher expression levels in the HPDE cell line compared to three other tumor cell lines (**Figures 9A, B**). This result is consistent with the results of the previous analysis. Furthermore, the high risk genes (*FAM83A-AS1*, *LINC02257*, and *TMEM105*) also exhibit higher expression levels in the three tumor cell lines (**Figures 9C–E**). In summary, the experimental results from qPCR are consistent with database analyses, which further confirms the accuracy and stability of the model lncRNAs at the biological level.

## Discussion

Previous studies have shown that LLPS has a significant impact on the formation and regulation of lncRNA, and there is a mutual relationship between TME and LLPS (29, 30). Additionally, the regulatory role of lncRNAs in various cancers has been well confirmed (31, 32). Based on these insights, this study focuses on lncRNAs as the research subject and centers on the TME to investigate associations with LLPS to evaluate their potential for predicting prognosis in PC. To ensure the validity of our results, samples with OS of fewer than 30 days are excluded from the analysis (may result from complications such as infections or surgical issues). Moreover, this study employs a broad, integrative gene-set approach, rather than restricting analysis to a single biological process or pathway, to minimize missing potential lncRNAs. GeneCards website is used as the primary source of theme genes, relying on its curated literature evidence and comprehensive integrated gene information to enable precise screening and enhance reproducibility.

This study employs a combined analysis of COX regression and LASSO regression to select an ideal lncRNA combination. Based on the analysis results, the optimal combination is established, which includes six lncRNAs: *AL157392.3*, *BX284668.2*, *FAM83A-AS1*, *LINC02257*, and *TMEM105*. Notably, *AL157392.3* and *BX284668.2* in this combination act as protective genes, and their high expression is beneficial for patient prognosis. Furthermore, the validation of results from risk subgroup survival analyses across multiple databases confirms the effectiveness of this model. However, we observe no statistically significant differences in the Kaplan-Meier survival curves for clinical subgroups in the stage _III-IV_ group. This lack of significance may arise from the limited sample size in this subgroup (33). We also note that the AUC value for the first year in the ICGC cohort is 0.597, which is below 0.60. It is that this situation is influenced by the weaker early signals to some extent, given the evident increased trend in performance, with AUC values of 0.606 and 0.682 in the second and third years, respectively. Additionally, it is worth that clinical cox regression analysis and clinical characteristics ROC curves for risk subgroups also indicate that the model lncRNAs can serve as independent risk factors for patient prognosis. If sufficient samples become available in the future, the research team will further validate the results. Additionally, the analysis related to TMB values indicates that the differences in survival times among samples within the independent mutation subgroup are not statistically significant. This implies that variations in TMB values have no significant impact on the survival time of these samples. However, the combined analysis of mutation subgroups with risk subgroups reveals significant statistical differences, further highlighting the validity and accuracy of the model lncRNAs. Furthermore, the expression levels of the model lncRNAs across the four cell line groups are consistent with analysis results, providing biological evidence that supports the reliability of the results. The lncRNAs *AL157392.3*, *FAM83A-AS1*, *LINC02257*, and *TMEM105* have been previously shown to have a positive correlation with the progression of various cancers, which aligns with the results of this study (34–38). Among them, *FAM83A-AS1* has been demonstrated to play a regulatory role in TME-related signaling pathways such as PI3K, Hippo, and ERK (36). *LINC02257* has also been shown to influence the expression of *SERPINE1*, which participates in multiple TME signaling pathways, including MAPK and TGF signaling pathways (37). Additionally, In NPInter database (version 5.0), the protein partners of *LINC02257*, *BX284668.2*, and *FAM83A-AS1* are DCP1, EWSR1, and hnRNPA1, respectively, all of which are regulators implicated in LLPS processes. This case further indicates the potential of the model lncRNAs and provides a reference for subsequent research and exploration.

### Limitation

It is worth noting that, although the study validates the effectiveness of model lncRNAs from various angles, there are still several limitations inherent in the research. First, it should be noted that this study focuses on correlation and exploratory analyses, and qPCR is used to corroborate the expression-level findings. Biological mechanisms are not explored in this study. Undeniably, this study is retrospective in nature, which means that its results may be influenced by the intrinsic design of this research method (39–41). Additionally, It is also worth noting that the present dataset lacks data on resectability status and postoperative adjuvant modalities, such as radiotherapy and chemotherapy, which will influence the performance of the model to some extent. To enhance the evaluation of model lncRNAs performance, researchers attempt to include more samples and data; however, corresponding lncRNAs expression information have not yet been obtained from gene matrices in existing databases. This is also the next direction for the research team, which will involve collecting more clinical samples and supplementary data to further explore and validate the risk signature. In summary, this study explores the association between the TME and LLPS-related gene sets to construct an lncRNA-based prognostic model for PC. The lncRNAs identified from preliminary analyses show potential as predictors of prognosis for PC patients.

## Conclusion

In conclusion, through multi-database analysis, this study explores the associations between the TME and LLPS-related gene sets to establish a lncRNA-based, potentially independent prognostic factor for PC.

## Data Availability

The datasets presented in this study can be found in online repositories. The names of the repository/repositories and accession number(s) can be found in the article/**Supplementary Material**.
